# MoreAir: A Low-Cost Urban Air Pollution Monitoring System

**DOI:** 10.3390/s20040998

**Published:** 2020-02-13

**Authors:** Ihsane Gryech, Yassine Ben-Aboud, Bassma Guermah, Nada Sbihi, Mounir Ghogho, Abdellatif Kobbane

**Affiliations:** 1 TICLab Research Laboratory, International University of Rabat, Rabat 11103, Morocco; yassine.benaboud@uir.ac.ma (Y.B.-A.); bassma.guermah@uir.ac.ma (B.G.); nada.sbihi@uir.ac.ma (N.S.); 2 ENSIAS, Mohammed V University in Rabat, Rabat 11103, Morocco; abdellatif.kobbane@um5.ac.ma

**Keywords:** urban air pollution, particulate matters, IoT, mobile sensing, pollution monitoring, Machine Learning, random forest, SVR, geographical information systems

## Abstract

MoreAir is a low-cost and agile urban air pollution monitoring system. This paper describes the methodology used in the development of this system along with some preliminary data analysis results. A key feature of MoreAir is its innovative sensor deployment strategy which is based on mobile and nomadic sensors as well as on medical data collected at a children’s hospital, used to identify urban areas of high prevalence of respiratory diseases. Another key feature is the use of machine learning to perform prediction. In this paper, Moroccan cities are taken as case studies. Using the agile deployment strategy of MoreAir, it is shown that in many Moroccan neighborhoods, road traffic has a smaller impact on the concentrations of particulate matters (PM) than other sources, such as public baths, public ovens, open-air street food vendors and thrift shops. A geographical information system has been developed to provide real-time information to the citizens about the air quality in different neighborhoods and thus raise awareness about urban pollution.

## 1. Introduction

The global air pollution crisis is a major issue that threatens our planet. It has several adverse effects on human health and the living ecosystem in general. In fact, State of Global Air (SOGA) has shown that exposure to air pollution reduces life expectancy by 20 months on average worldwide, and by 18 months in North Africa and Middle East [1]. The main types of air pollutants are: gaseous pollutants (e.g., carbon dioxide (${\mathrm{CO}}_{2}$), carbon monoxide (CO), Sulphur dioxide(${\mathrm{SO}}_{2}$), Ozone ($O_{3}$), nitrogen oxide (NO) and nitrogen dioxide (${\mathrm{NO}}_{2}$)) and a complex mixture of solid and liquid droplets called particulate matters (PM) (e.g., ${\mathrm{PM}}_{2.5}$, ${\mathrm{PM}}_{10}$) [2,3]. PM cause many respiratory diseases, such as asthma, chronic obstructive pulmonary disease and respiratory infections.

Citizens are generally not aware of the damages caused by air pollution, and are not informed about the spatial distribution of air quality. This is particularly true in low- and middle-income countries. Indeed, capturing the spatial variability of air pollution in cities requires a dense deployment of air quality monitoring stations [4,5]. The latter allow for precise measurements of air pollution; reliability of the measurements is ensured by applying standard procedures for instrument calibration, data collection and post-processing. An example of a network of such stations is the Automatic Urban and Rural Network (AURN), which is the UK’s largest automatic monitoring network and is the main network used for compliance reporting against the Ambient Air Quality Directives [6]. However, the deployment and maintenance of a high number of these fixed air monitoring networks are very expensive. One can expect at least $10K per station, excluding installation and maintenance costs[7]. Furthermore, these monitoring stations are generally not located in regions where anthropogenic activities and populations are concentrated; roadsides and major traffic congestion areas are also often very far from the measuring stations, which may significantly affect the accuracy of the pollutants’ spatial distribution estimation in urban areas [8]. To address these challenges, attention has recently been redirected towards using small low-cost sensing units. In [9], McKercher et al. compare costs of portable gaseous air pollution monitoring devices, which range from $180 to $4900. While these units can, when densely deployed, provide more data, their accuracy is generally lower compared to that of fixed air monitoring stations [10]. This accuracy issue has been investigated in various studies, e.g., [11]. Adriana et al. used a combination of stationary/fixed and smart mobile pollution sensors that were carried on a daily basis by citizens, and showed that the mobile units detected a significantly higher level of NO2 concentrations, which were sometimes between three to five times higher than those measured by the passive-static monitoring tubes [12]. Another study was done by McKercher et al., where in addition to a fixed station, [13] presents the smart citizen kit (SCK) which is a low-cost, portable air quality monitor, capable of measuring CO, ${\mathrm{NO}}_{2}$, temperature, relative humidity, light intensity, and sound levels. The sensors were deployed on bicycles going in loops on a predefined path in the city of Lubbock, Texas. The authors of [14] introduce the smart personal air quality monitoring system (SPAMS), which collects $O_{3}$, ${\mathrm{NO}}_{2}$, CO, and ${\mathrm{PM}}_{2.5}$, temperature, and relative humidity, while on the move. They investigated the calibration and data validation before presenting some results of their measurement campaign in different spots of the city of Chennai, India. Similarly, Ref. [15] presents LILI-1, a low-cost solution for the monitoring of $O_{3}$, ${\mathrm{NO}}_{2}$, ${\mathrm{PM}}_{10}$, ${\mathrm{PM}}_{2.5}$, temperature, relative humidity, and atmospheric pressure; the issues related to the choice of the hardware, calibration, deployment strategies and data evaluation are addressed.

Another challenge facing air quality forecasting is the large number of factors that influence air pollutants’ concentrations. Two of the most known factors to impact air quality are meteorological and road traffic-related features [16,17]. In [18,19], Banarjee et al. studied the impact of meteorological features, including temperature, humidity, and wind on the concentration of pollutants in India. In [20], pressure and isolation were added as features to find the best data mining model for air pollution forecasting. As to the impact of road traffic on pollution, the 2012 air quality assessment [21] indicates that in France 56% of the nitrogen dioxide in the air is caused by transportation. In [22], Wallace et al. used the Integrated Model of urban Land-use and Transportation for Environmental Analysis to estimate emission and concentrations of NOx from traffic sources in the Hamilton census metropolitan area . The results showed a prominent triangle area of high pollution, which is defined by major roads and highways along the Hamilton Harbor during peak hours. The number of studies using machine learning to model air quality has been increasing dramatically over recent years. However, only very few studies have been carried out in low-income countries [23]. Furthermore, despite the high number of these studies, few of these used both traffic and meteorological features at the same time to infer air quality concentrations via machine learning. Most existing papers would only use meteorological features as predictors. In this paper, we introduce the MoreAir urban air pollution monitoring system. The main contributions of the paper are as follows:

- We propose a novel approach to designing low-cost air pollution monitoring systems, which consists of a combination of (i) a novel sensor deployment strategy, based on mobile and nomadic sensors as well as on a prior medical survey, (ii) machine learning to perform model-based interpolation, and (iii) the Internet of Things to provide the users with real-time air quality data.
- We propose a methodology to build datasets which include pollutants’ concentrations, meteorological conditions, traffic-related features, and small-scale details of the different neighborhoods including social activities.
- We compare three machine learning models to predict pollutants’ concentrations: Multiple Linear Regression (MLR), Support Vector Regression (SVR) and Random Forest (RF). We show that RF and SVR better describe the non-linear impact of traffic flows and meteorology on PM concentrations.
- We show that in many disadvantaged neighborhoods in Morocco, social activities have a higher impact on air quality than road traffic.

The paper is structured as follows. Section 2 describes the Internet of Things (IoT) Platform which we have developed to collect data. Section 3 describes the sensor deployment strategy based on a prior medical survey. Section 4 provides preliminary results and an analysis of the main sources of air pollution found in the city of Rabat and outskirts. Section 5 presents some preliminary results on the use of machine learning algorithms for predicting air quality. Section 6 describes the developed open source GIS. Finally, concluding remarks and possible extensions of this work are provided in Section 7.

## 2. IoT Platform

**Figure 1:**
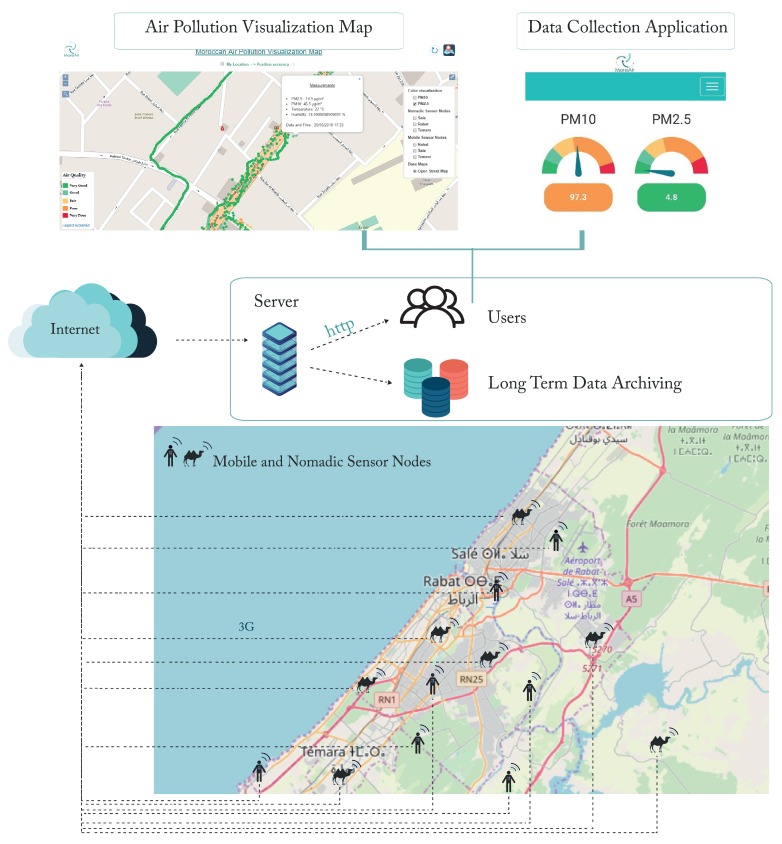
General architecture of the IoT platform.

In this section, we describe the Internet of Things (IoT) platform developed to collect data. Data collection was carried out using low-cost sensing units which we have developed in our laboratory. One of the main goals of this initiative is to make the cost of monitoring air pollution affordable, especially in low and middle-income countries. Figure 1, illustrates the developed platform.

### 2.1. Sensor Node Development

We developed two categories of sensor nodes:

- MoreAir AQ-N: Nomadic sensor nodes (represented by a camel icon in Figure 1) with a better power supply but settled in one specific location during the collection process (the duration varies from 1 week to several months).
- MoreAir AQ-M: Mobile sensor nodes (represented by the man icon in Figure 1) that operate on high capacity batteries but has a lower energy autonomy (around 24 h); the mobile sensor nodes are used to collect data while on the move to capture local concentrations variability and detect hot-spots.

Low-cost, easy assembly and market availability are the three main criteria considered for choosing the components. In addition to the easy interfacing of the components, the total node price (for both categories) does not exceed $95 making the duplication process of the sensors very affordable. In Table 1, other solutions in the market measuring concentrations of particulate matter have been compared according to their type and cost. It is shown that these solutions range from hundreds of dollars for hand-held monitors, to thousands of dollars for more sophisticated stations. Ref. [24] presents a more in-depth review of sensors for air quality monitoring sensors in the market

**Figure 2:**
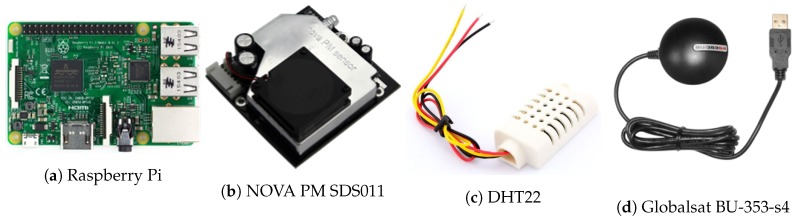
Hardware used in the sensor nodes.

The hardware used in the developed sensing nodes is composed of only low-cost off-the-shelf components, which are listed below:

- Computing Unit: a Raspberry Pi was used because it has sufficient computing power for data collection, and is easy to use and interface with the other sensors (Figure 2a).
- Sensing Unit: the concentrations of ${\mathrm{PM}}_{10}$ and ${\mathrm{PM}}_{2.5}$ were sampled using the NOVA PM SDS011 sensor (Figure 2b), which is a digital sensor based on a laser scattering principle for a reliable, accurate and stable output quality. Moreover, a temperature and humidity sensor (DHT22) was added to have a more precise information about the humidity and temperature in the monitored area (Figure 2c).
- Tracking Unit: each mobile sensor node is equipped with a Globalsat BU-353-s4 GPS to provide an estimate of its location (Figure 2d). Nomadic sensor nodes are not equipped with GPS.
- Transmission Unit: each sensor node is equipped with a USB 3G/4G modem to transmit the collected data in real time to our servers. For our application, Long Range (LoRa) and similar technologies are more suitable (and less expensive). However, in Morocco, the use of the frequencies associated with these technologies is still not open to the public. In addition to the flexibility of the 3G/4G technologies (especially for mobile sensors), the prices of cellular plans in Morocco are very low ($4 a month per sensor is more than enough for our application).
- Power Unit: each mobile sensor node is powered by 20,000 Ah Battery, and each nomadic sensor node is equipped with a 7 Ah battery and a solar panel.

A modular software was implemented in the sensor nodes to offer an abstraction of different functionalities allowing easy updates and integration. The main functionalities are:

- Data sensing: handles the data extraction, sampling and filtering processes.
- Data formatting: handles the pre-processing and the encapsulation of the data before transmission. This functionality is managed by a logical middleware between the nodes and the server.
- Data transmission: allows the sensor nodes to send data to the server over the Internet.

**Figure 3:**
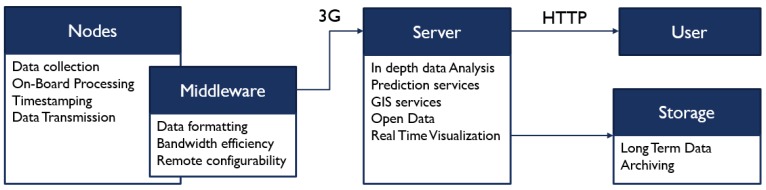
Diagram of the IoT platform’s components and their roles.

Figure 3 summarizes the roles of the different components of the platform and how they are connected.

### 2.2. Sensor Node Evaluation

**Figure 4:**
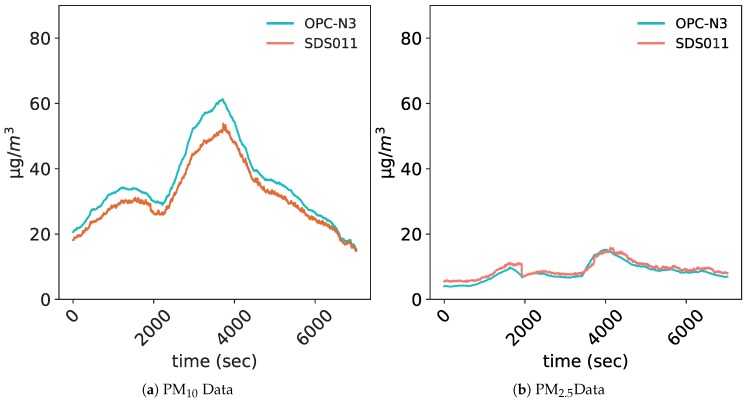
Particulate matter data collection runs.

The downside of using low-cost sensors is the reduced accuracy of the measurements. The experimental process to evaluate the low-cost PM sensors needs to respect several aspects; including the operational stability under different meteorological conditions, the precision of sensors in terms of reproducibility between units of the same sensor model (so-called intramodel variability) and the performance of sensor operation in air with high relative humidity. In [32], Badura et al. evaluated the performance of multiple low-cost sensors by considering all the above-mentioned aspects. It was shown that the SDS011 is accurate in terms of reproducibility between units, and that it is reliable for detecting elevated PM concentration events or indicating PM “hot-spots”. A similar study was conducted by Lieu et al. in [33] to evaluate the ${\mathrm{PM}}_{2.5}$ data quality of the NOVA PM SDS011; the experiment was conducted over a period of nearly four months, in which the sensor was quite stable, and no obvious sensor errors have been observed. To further validate the choice of our PM-sensor (namely the NOVA PM SDS011), we carried out a small experiment at our University. We placed the sensor next to the more sophisticated OPC-N3 optical particle counter from Alphasense [34] (The latter’s data quality has already been evaluated and validated in different studies [14,35,36]). Three measurement runs using both sensors were conducted. The data is collected for 2 h per run with a moving average of 5 min. Figure 4 shows one of the measurement runs conducted in our laboratory.

We notice that our reference sensor is more sensitive to variations in concentrations than the NOVA PM SDS011 sensor, but the difference is not very important. We use the coefficient of determination ($R^{2}$) and the mean absolute error (MAE) as indicators to evaluate the quality of the data collected by our sensor in comparison with the reference sensor. Table 2 shows the values of the indicators for both ${\mathrm{PM}}_{2.5}$ and ${\mathrm{PM}}_{10}$ data.

With these $R^{2}$ values, we can safely conclude that the low-cost chosen sensor’s data fit well the reference sensor’s data. In addition, the concentrations of ${\mathrm{PM}}_{2.5}$ and ${\mathrm{PM}}_{10}$ measured in urban areas during high activity hours can go as high as 180 μg/m^3^ meaning that a mean absolute error of 3.97 (resp. 1.03) is very low. These two indicators show that the performance of the chosen low-cost sensor is very good, thus making it a viable option to use in our project.

## 3. Deployment Strategy and Data Collection

### 3.1. Description of the Selected Sites

Our air pollution monitoring system is designed to measure outdoor air pollution caused by both diffuse and point sources of pollution. Thus, the sites selected for this initial study represent, when combined, different types of pollution sources and diverse neighborhoods. These two aspects are necessary to build reliable machine learning models to predict air quality in urban areas.

The selection of sites was primarily based on the medical data that we have collected at the Rabat children’s hospital. The areas where children admitted to the emergency department live have been selected for air quality monitoring. The areas turned out to have a low socio-economic status, according to the perceptions of the general public. Such neighborhoods have narrow streets filled with markets, vendors, thrift shops characterized by a very low traffic volume. This may explain why on such neighborhoods the pollutants’ concentrations are more affected by the human activity than by traffic. To compare the air quality in these areas with that in other areas of the city, we have also included neighborhoods of a higher socio-economic status in our data collection campaigns. During these campaigns, we have collected measurements of pollutants’ concentrations and identified possible sources of pollution in the neighborhood.

**Figure 5:**
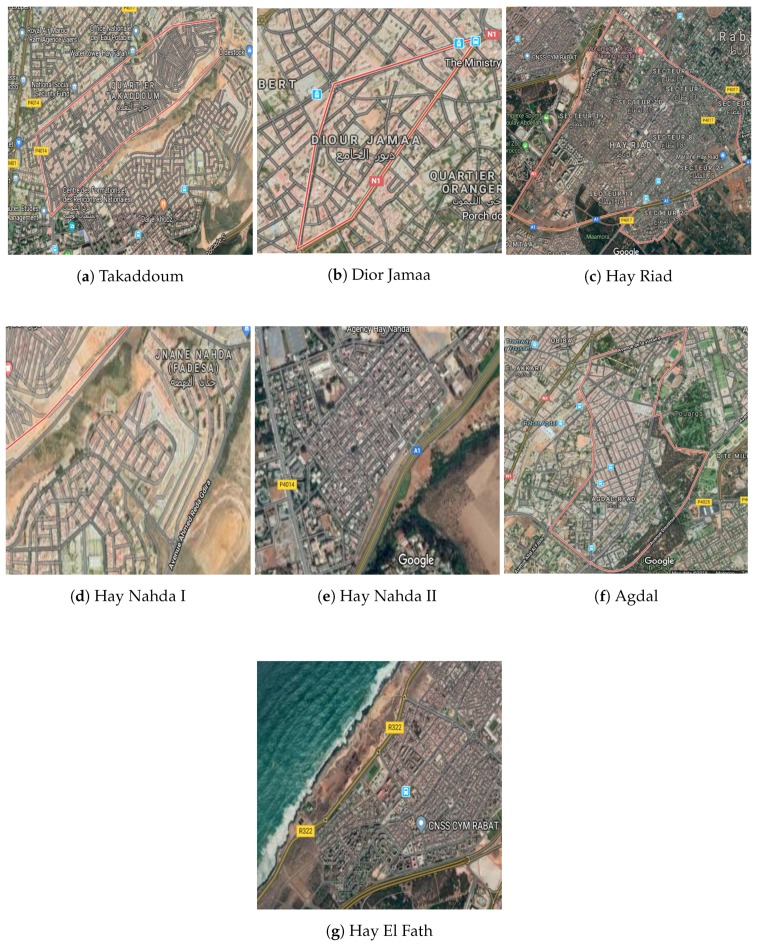
Location of the selected sites on Google Maps.

So far, we have collected data in the following seven neighborhoods: Takaddoum Figure 5a, Dior Jamaa Figure 5b, Hay Riad Figure 5c, Hay Nahda I Figure 5d, Hay Nahda II Figure 5e, Agdal Figure 5f, and Hay El Fath Figure 5g.

Takaddoum is a district, characterized by high building and population densities. It has one main road (Avenue Al Haouz). The latter facilitates access to all kind of public transportation, which makes it often very busy. The remaining parts of the district consist of housing, commercial spaces, and very dynamic social activities. Schools, hospitals, youth houses, and a few green spaces for children are also found in this district. During our data collection campaigns, we monitored air quality in the numerous narrow streets of the district. These streets host open street food vendors, street clothes vendors, thrift shops, repair shops, traditional baths and traditional ovens.

**Figure 6:**
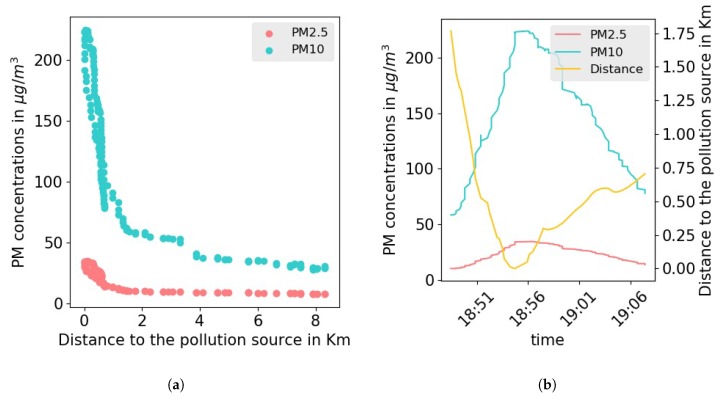
PM concentrations vs distance to the area of interest.

The activities found in such locations can negatively affect the air quality. For instance, public baths and ovens generate large amounts of pollutants from burning wood and coil. These sources of pollution have been identified by monitoring the levels of PM concentrations while walking in the narrow streets of Takaddoum. Whenever air quality measurements increase drastically in an area, we explored this area further to identify the sources of pollution. An example of such a case was observed in a small area that hosts open-air thrift shops and street food vendors. The observed increase of the ${\mathrm{PM}}_{10}$ (resp. ${\mathrm{PM}}_{2.5}$) during the collection campaign were further validated by numerical analysis of the collected data. Figure 6 shows a strong negative correlation between the distance to the center of the area of interest (thrift shops and food vendors) and the concentration of ${\mathrm{PM}}_{10}$ (resp. ${\mathrm{PM}}_{2.5}$). The visual correlation was validated by a correlation coefficient of −0.78 (resp. −0.74) with a *p*-value in the order of $10^{-33}$ (resp. $10^{-23}$). The same observations were consistently made and tested on different days and for different sources of pollution in all the studied neighborhoods.

Using this approach, in Takaddoum we have identified the following five main sources of pollution:

- Street vendors: these include the large number of vendors of fresh fruit and vegetables, and vendors of cooked food using mostly open-air charcoal stoves. Another common type of street vendor found in Takaddoum’s streets are open-air thrift shops and clothes vendors.
- Repair shops: Some streets of Takaddoum host many repair shops whose activities mainly take place outdoor. These include welding, carpentry, car and bicycle repair and servicing.
- Open sewage impoundments, canals and conveyance systems: These do not only cause foul odor but also increased mold in the area.
- Traditional baths and ovens: public bathhouses, known as hammams or Moroccan baths, are a living heritage sustained through many centuries and are still considered to be a strong tradition in Morocco. The number of operational hammams in Morocco, using the traditional heating system, varies between 6000 and 10,000 [37]. Public ovens (or communal ovens) are also still used by many people, particularly for baking bread. Often, the public bath and oven are co-located to share the same heating system. Studies indicate that each public bath-oven unit consumes between one and two tons of wood per day for both space and water heating [37,38,39]. The burning of such a large amount of firewood causes the release of large amounts of carbon dioxide and other air pollutants.
- Building materials: in many countries, the use of certain materials, like sheet metal, is forbidden because of the numerous damages it has on human health [40]. The serious diseases related to inhaling low doses of the asbestos found in sheet metal include pulmonary fibrosis, bronchopulmonary cancers, and pleura or abdominal cavity [41,42,43]. All asbestos varieties are currently classified as carcinogens by the International Agency for Research on Cancer (IARC) [44]. Yet, this material is still used for construction in certain areas.

The second and third sites selected are Hay Riad and Agdal which are among the opulent neighborhoods of the city. Data collection was performed on “Avenue Annakhil” and “Avenue de France”, respectively. Avenue Annakhil has a main road, often crowded especially during rush hours. As to Avenue de France, it is part of the Agdal-Ryad district of the municipality of Rabat; it has a small road compared to the one in Avenue Annakhil, but it is still considered a dual carriageway with a Tramway line in the middle. In these two neighborhoods we find administrations, offices, a few restaurants/cafes and shops, and apartments on each side of the road. There are no open-air street vendors and no public baths and ovens in these neighborhoods. Therefore, we believe that the main persistent source of pollution in these neighborhoods might be road traffic. We recall that the most common transport means inside the city are cars, buses and minivans, motorcycles, Tramways, mini trucks and few big trucks.

The fourth site is Hay Nahda II, which consists mainly of residential areas of different socio-economic status. This is a quiet neighborhood except for a very few street vendors and a few car repair shops. Hay Nahda I and the part of Hay El Fath where data was collected share the same characteristics as Hay Nahda II. The only additional feature that distinguishes Hay El Fath from these two neighborhoods is its proximity to the ocean.

### 3.2. Sensor Deployment Strategy

Air pollution is traditionally monitored using the so-called active technique, which consists of equipping each measurement site with one or more highly reliable stations measuring continuously and automatically one or more pollutants. Morocco has 29 of such stations placed in 15 different cities and 3 mobile stations [45]. However, such stations entail high investment and maintenance costs, which explain why many of these stations are often non-operational for long periods of time and leads to the loss of important data. Furthermore, even though these stations provide highly accurate data at the measurement sites, because of their small number due to high costs, they cannot capture the spatial variability of pollutants concentrations, particularly in urban areas. Furthermore, it is generally difficult to install such stations in urban areas, especially in neighborhoods characterized by dynamic daily activities, which have significant effects on urban pollution coverage. Our approach consists of using a hybrid deployment strategy based on using low-cost nomadic and mobile sensor nodes, and machine learning to perform model-based spatial interpolation. Nomadic sensor nodes are installed, for a specific period of time, on the facades of buildings with a street view, in different neighborhoods experiencing different social activities, and near schools and hospitals. Mobile sensor nodes are, on the other hand, carried by people or vehicles to allow data collection at many locations. The two types of sensor nodes complement each other. Nomadic (mobile) sensor nodes provide a high (low) temporal coverage but a low (high) spatial coverage. It is also worth pointing out that mobile sensor nodes are more appropriate where vandalism is an issue.

**Figure 7:**
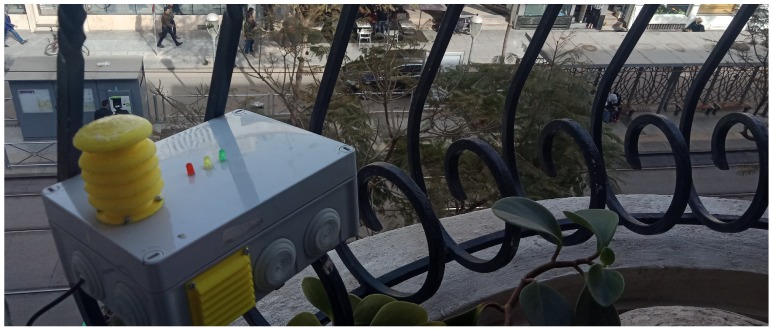
Nomadic sensor node installation.

In Takaddoum, Dior Jamaa, Hay Riad, and Hay Nahda II, mobile sensor nodes were carried by volunteering students on foot or in vehicles. In Hay Nahda I, Hay El Fath and Agdal, we used nomadic sensor nodes, which were placed on the facades of buildings at different heights. Since the MoreAir project focuses on health and aims to raise awareness among citizens, we considered lower height levels for monitoring when using mobile sensors, e.g., the breathing zone of 1.5 m. However, according to the Central Pollution Control Board (CPCB), the monitoring should be done outside the zone of influence of sources located within the designated zone, including traffic and any other pollution source. Thus, the height of the inlet must be 3–10 m above the ground level. Therefore, for nomadic sensors the following measuring heights have been chosen: 6 m for Hay Nahda I, and 11 m for Agdal and Hay El Fath, to assess the impact of height on pollutants concentrations. Figure 7 shows a nomadic sensor node at the window of an apartment in Agdal.

### 3.3. Air Quality Data Collection

For each nomadic sensor node installation, data collection was carried out over at least one week. For mobile sensor nodes, data was collected every day in different neighborhoods for a continuous one-hour using mobile sensor nodes and a human carrier, often in the afternoon around 17:00, over a period of two months (May and June 2018). The sensors were configured to take measurements every five seconds.

The data set constructed from the collected measurements consists of:

- ${\mathrm{PM}}_{10}$ and ${\mathrm{PM}}_{2.5}$ concentrations records in μg/m^3^,
- Temperature and relative humidity in °C and % respectively,
- GPS data (namely latitude, longitude and altitude); only for mobile sensor nodes,
- Timestamp associated with each measurement.

**Figure 8:**
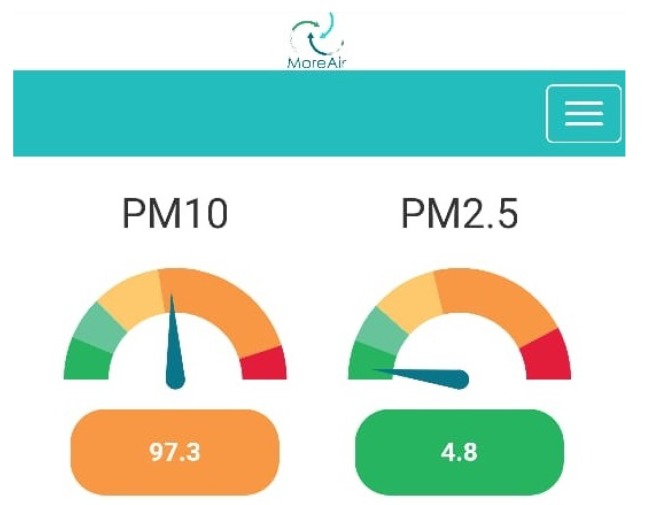
Chart representing the concentrations of ${\mathrm{PM}}_{10}$ and ${\mathrm{PM}}_{2.5}$ in μg/m^3^ as shown by the mobile app.

We have also designed a mobile application (see Figure 8) to show in real time the pollution measurements collected by the sensor nodes. This is particularly useful for the mobile sensor nodes. Indeed, this can help the human carrier identify, on the go, areas of high pollutant concentrations and thus explore them further by spending more time in these areas to check whether or not the high concentrations are transitional.

### 3.4. Pre-Processing

**Figure 9:**
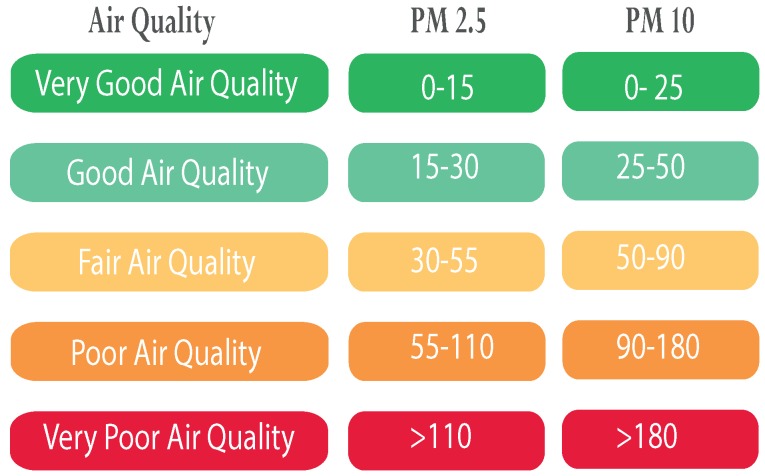
European air quality Index (${\mathrm{PM}}_{10}$ and ${\mathrm{PM}}_{2.5}$ in μg/m^3^).

**Figure 10:**
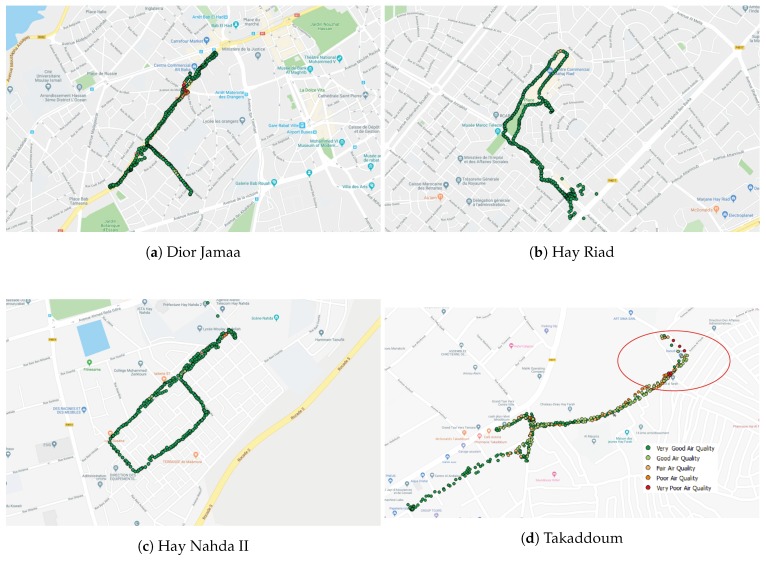
QGIS-based visualization of some of the measurements collected with mobile sensor nodes.

To monitor and control air quality, the European Union has defined the European Air Quality Index which is based on the values of several pollutants’ concentrations [46]. We base our color coded visualization of pollutants’ concentrations on this index; see Figure 9. The collected data is imported on Quantum GIS (QGIS) which not only allows visualization of the data and the locations of the pollution sources (see Figure 10) but also to evaluate the relevant features needed for the spatial modeling of air pollution using machine learning.

When there were missing data, which was the case with the temperature and humidity measurements, we used a linear interpolation to impute the missing values. After cleaning the data set by removing irrelevant data and outliers, we converted the time stamp into a more intelligible format.

## 4. Descriptive Data Analysis

In this section, we report on the analysis of the data collected by the mobile sensor nodes in four neighborhoods, on different days but over approximately the same time period. We also report on the data collected by the nomadic sensor nodes in two neighborhoods over a one-month period.

**Figure 11:**
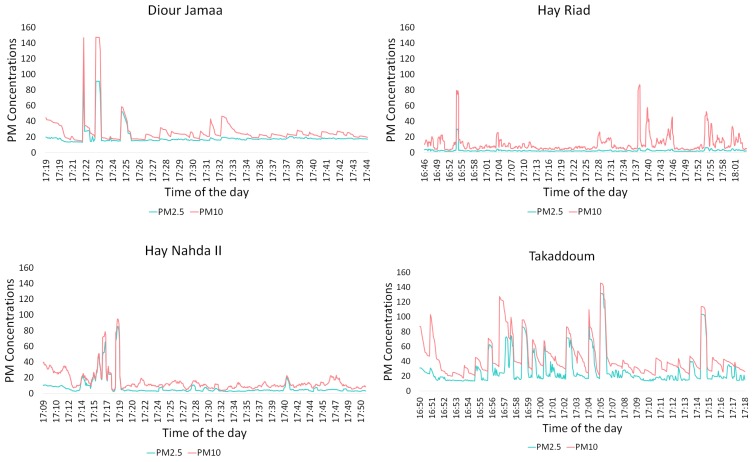
${\mathrm{PM}}_{10}$ and ${\mathrm{PM}}_{2.5}$ measurements in μg/m^3^ recorded in four neighborhoods.

Figure 11, depicts the measurements of the concentrations of ${\mathrm{PM}}_{10}$ and ${\mathrm{PM}}_{2.5}$ obtained with mobile sensor nodes. It is shown that these concentrations generally do not exceeded the limit values (50 μg/m^3^ and 30 μg/m^3^ respectively). However, high concentrations were observed when the sensor node is close to a specific pollution source; see peaks in Figure 11 and Table 3.

In Hay Riad, the recorded ${\mathrm{PM}}_{10}$ concentrations exceeded the limit only in the presence of some vehicles, especially near a roundabout. On the other hand, ${\mathrm{PM}}_{2.5}$ remained low and stable.

In Diour Jamaa, the lowest values of the recorded ${\mathrm{PM}}_{10}$ and ${\mathrm{PM}}_{2.5}$ concentrations were of 14.9 μg/m^3^ and 13 μg/m^3^ respectively. The data was collected while walking very close to a two ways road; so we suspect that the main source of pollution is traffic. The observed two peaks of ${\mathrm{PM}}_{10}$ concentration that exceed 140 μg/m^3^ were both recorded near open street food vendors.

In Hay Nahda II, PM concentrations only increased when we walked past a mechanical repair shop. Otherwise, PM concentrations were low.

In Takaddoum, where most asthma patients came from, the recorded PM concentrations were higher than in the other neighborhoods. Furthermore, the variability of the concentrations while moving is high. This is due to the variety of pollution sources mentioned before (Hammams, thrift shops, open street vendors). The highest concentrations seem to be often associated with either public baths and ovens or street vendors.

**Figure 12:**
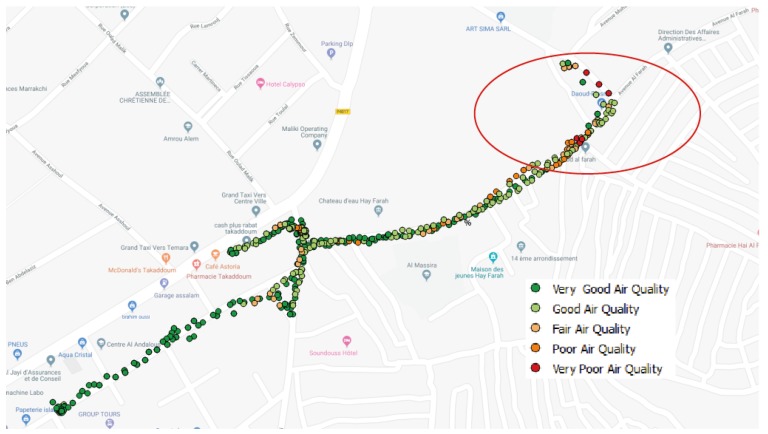
The high PM concentrations recorded in Hay Al Farah, Takaddoum.

Another observation that caught our attention during data collection in Takaddoum is that the highest concentrations were recorded in Hay Al Farah; see the part circled in red in Figure 12 (800 μg/m^3^ for ${\mathrm{PM}}_{10}$ and 248 μg/m^3^ for ${\mathrm{PM}}_{2.5}$). Hay Al Farah is a small but very crowded street mostly occupied by street vendors, making it very difficult for cars to pass through it. Furthermore, building density is high which might be responsible for the stagnation of pollutants in the air.

According to the preliminary findings of our data collection campaigns, one suspects that the high rates of PM’s concentrations in the studied disadvantaged neighborhoods are mainly due to public baths and ovens, open-air thrift shops and street food vendors, as PM concentrations rocketed in the vicinity of these sources, and that road traffic is not the main contributor to air pollution in these neighborhoods. It is worth pointing out that street food vendors are not only responsible for the emission of PM’s, but also for the emission of VOCs and SVOCs as a result of cooking meat, and for the emission of CO and NO as a result of using charcoal fire [47]. Sensors of some of these pollutants will be added to our sensor nodes in the future.

It is, therefore, fair to infer that the high incidence of respiratory diseases in Takaddoum, observed at the children’s hospital, may be due to the poor air quality in this neighborhood. This also confirms that our sensor deployment strategy, based on medical records, is efficient in identifying areas of poor air quality with a small number of low-cost sensors.

**Figure 13:**
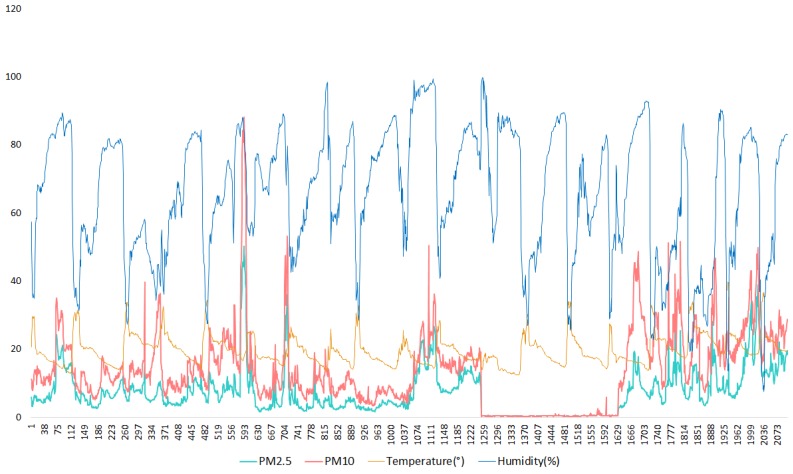
PM concentrations in μg/m^3^, recorded by a nomadic sensor in Hay Nahda I.

Figure 13, depicts the data collected from the nomadic sensor in Hay Nahda I over a one-month period. Averages over some time windows varying from 8 hours to 1 day are often used. However, in order to capture the variability of pollutants concentrations with a smaller temporal granularity while avoiding the outliers, caused by volatile sources of pollution, we applied 10 min averages to the air pollution measurements which were taken continuously every 5 s. The region where the measurements were taken is characterized as a small neighborhood, with no specific activities, no main roads, a few green spaces in its vicinity. Figure 13 also depicts some missing data over a period of two days. These data were removed in the prediction phase, but were left in the figure to show some of the issues that low-cost sensors may face. In this case, the loss of data was caused by the presence of insects on the PM sensor node; this issue was found also in other areas which are near green spaces. Since the sensor node was placed on a fixed position, the peaks registered did not vary much, unlike the ones obtained with mobile sensors. The observed peaks of ${\mathrm{PM}}_{10}$ and ${\mathrm{PM}}_{2.5}$ concentrations did not exceed 90 μg/m^3^ and 50 μg/m^3^ respectively.

**Figure 14:**
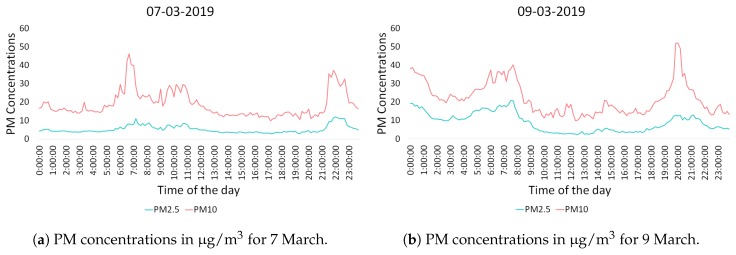
PM concentrations for two random days in the neighborhood of Agdal.

In the bigger neighborhood of Agdal, PM concentrations obtained with a nomadic sensor node were higher compared to those obtained at Hay Nahda I. Furthermore, since the sensor was placed near road traffic, the pollutants concentrations are shown to be often high during and around rush hours; see Figure 14.

## 5. Machine Learning-Based Modeling

One of the main objectives of MoreAir is to use machine learning to develop models that can predict the concentrations of PM based on the collected pollution data and exogenous features that are known to impact pollutants’ concentrations. The traditional methods used for air quality prediction are Linear Regression [48], auto-regressive integrated moving average (ARIMA) [49], and Kalman filtering (KF) [50]. These models are incapable of handling non-linearity. Furthermore, the models should include both endogenous and exogenous data. For example, traffic and weather-related features should be included in the prediction models. In this work, we compare three models, namely the traditional Multiple Linear Regression, Support Vector Regression and Random Forest. To build these models, feature engineering is of paramount importance. These features should be derived from phenomena that are known to impact air quality, which are meteorological conditions, road traffic, land use, building types and densities, and socio-economic activities. The dataset needed to build these models should consist of a large number of examples of input-output pairs where the input is the feature vector and the output is the concentration of a pollutant. One model per pollutant should be built. The methodology used to collect data to obtain different examples of the feature vector is as follows:

- Meteorological data is obtained from the temperature and humidity sensor of the sensor nodes, along with *meteoblue*, a website that gives open access to meteorological data .
- Traffic-related data is extracted from Google Traffic, as described in [51], which periodically captures Google Traffic maps as images and then applies image processing to extract the level of congestion on the main roads of the city.
- Features related to land use, building types and densities are extracted, using QGIS, from a 3D map of the city of Rabat provided by the city’s Urban Agency.
- Data on socio-economic activities are obtained by visual investigation of the different areas in which data collection was performed (localization of pollution sources such as hammams, public ovens, street vendors, etc.).

The building of the dataset is an ongoing work. Indeed, for the machine learning models to generalize well, the dataset must include diverse meteorological conditions; thus, the data collection must cover the four seasons.

While building our dataset, we have tested our approach on data gathered by one nomadic sensor node placed in the neighborhood of Agdal over a period of one month. Since only one sensor node is considered, we skipped the use of spatial data and only kept meteorological components and traffic to predict the air quality measured by the nomadic node.

We applied a 10-min average to the measurements of ${\mathrm{PM}}_{10}$ and ${\mathrm{PM}}_{2.5}$ which were initially taken every 5 s. For the meteorological features, we use temperature and humidity measurements that were also obtained with the nomadic sensor node.

For the traffic data set, data collection was performed using a novel method that consists of exploiting Google Traffic maps using image processing [51]. We first started by selecting the area of interest, which includes the roads close to the nomadic sensor placement (a distance of 2 m), and launched an automatic program that obtains screen captures of the traffic map each 5 min. Afterwards, by applying basic image processing tools, relevant traffic information is extracted. The state of traffic is represented by a categorical variable, displaying 4 levels of congestion ( Brown: high traffic congestion, red: congestion, orange: less traffic congestion and green: fluid traffic). Thus, we create multiple dummy variables to represent the categorical variable (i.e., traffic) and be able to apply regression analysis. Given the difference between data units, all data are normalized using Equation (1).

(1) $$x_{ij}^{\prime }=\frac{x_{ij}-min_{j}}{max_{j}-min_{j}}$$

where $x_{ij}$ is the relevant data for row *i* and parameter *j*, $x_{ij}^{\prime }$ is the modified data that falls into the scale [0,1], $min_{j}$ is the minimum of variable *j* and $max_{j}$ is the maximum of variable *j*.

The metric $\left(R^{2}\right)$ and the root mean square error (RMSE) have been calculated using Equations (2) and (3), [52,53] .

(2) $$R^{2}={\left[\frac{1}{n}\frac{\sum_{i=1}^{n}\left[\left(P_{i}-\bar{P}\right)-\left(O_{i}-\bar{O}\right)\right]}{\sigma_{p}\sigma_{o}}\right]}^{2}$$

(3) $$RMSE={\left(\frac{1}{n}\sum_{i=1}^{n}{\left[P_{i}-O_{i}\right]}^{2}\right)}^{\frac{1}{2}}$$

where *n* is the number of observations, $O_{i}$ is the observed parameter, $P_{i}$ is the calculated parameter, $\bar{O}$ is the mean of the observed parameter, $\bar{P}$ is the average of the calculated parameter, $\sigma_{o}$ is the standard deviation of the observations and $\sigma_{p}$ is the standard deviation of the calculations.

A separate prediction is conducted for each pollutant (${\mathrm{PM}}_{10}$ and ${\mathrm{PM}}_{2.5}$), using Multiple Linear Regression (MLR), Support Vector Regression(SVR) and Random Forest. The results of these prediction algorithms are presented in Table 4.

The first model used for prediction is Multiple Linear Regression, which is a simple algorithm known for its low complexity, easy implementation and interpretability. The MLR assumes the following relationship between the explanatory (independent) variables and response (dependent) variable,

(4) $$Y=\beta_{0}+\beta_{1}X_{1}+\beta_{2}X_{2}+\ldots +\beta_{p}X_{p}+\epsilon$$

where *Y* is the dependent variable, $X_{i}$ is the ith explanatory variable (or feature), ($\beta_{0}$,..., $\beta_{p}$) are the MLR parameters, and ϵ is the model’s residual. By using this algorithm, $R^{2}$ did not exceed 0.22 and 0.42 for ${\mathrm{PM}}_{10}$ and ${\mathrm{PM}}_{2.5}$ respectively. Hence, this model fails to capture the relationship between the PM concentrations and the predictors, which is why we investigated two non-linear models, namely Support Vector Machine for Regression (SVR) and Random Forest.

SVR is a powerful neural network-based model which relies on kernel functions to provide the best fit to observed data. It aims to map a high-dimensional feature space to the considered output. Different kernel functions can be adopted [54]. In this work, we consider a Gaussian Kernel function. Hence, the prediction takes the following form

(5) $$Y=\sum_{i=1}^{m}\theta_{i}\exp\left(\frac{-||X-x_{i}{\left|\right|}^{2}}{\gamma }\right),$$

where $X=[X_{1},\ldots ,X_{p}]$, $x_{i}$ is the value of the feature vector that corresponds to the ith observation, *m* is the number of observations, γ is a tuning parameter, and the $\theta_{i}$’s can be computed based on the cost function by evaluating the difference between the predicted values and the real values of pollutants’ concentrations, to a threshold ϵ [55], determined through cross validation. Compared to MLR, SVR improves air quality prediction, with an $R^{2}$ equal to 0.39 and 0.47 for ${\mathrm{PM}}_{10}$ and ${\mathrm{PM}}_{2.5}$ respectively.

Random Forest is one of the most popular Ensemble Learning techniques. Random Forest is based on an ensemble of decision tree predictors. It uses a modified tree learning algorithm which selects a random subset of the available features (feature bagging) to reduce the correlations between the trees; for a dataset with p features, $\sqrt{p}$ features are used in each split. Moreover, each decision tree is trained on a different set of randomly chosen observations obtained using the Bootstrap method. Random Forest significantly outperforms both MLR and SVR, with $R^{2}$ values of 0.57 and 0.63 for both ${\mathrm{PM}}_{10}$ and ${\mathrm{PM}}_{2.5}$ respectively.

In Table 4, it is shown that Random Forest for both of pollutants gives the minimum error and the best $R^{2}$, followed by Support Vector Regression and then Multiple Linear Regression. The prediction accuracy of these algorithms should increase once more data are collected.

## 6. Moroccan Urban Air Quality Map

**Figure 15:**
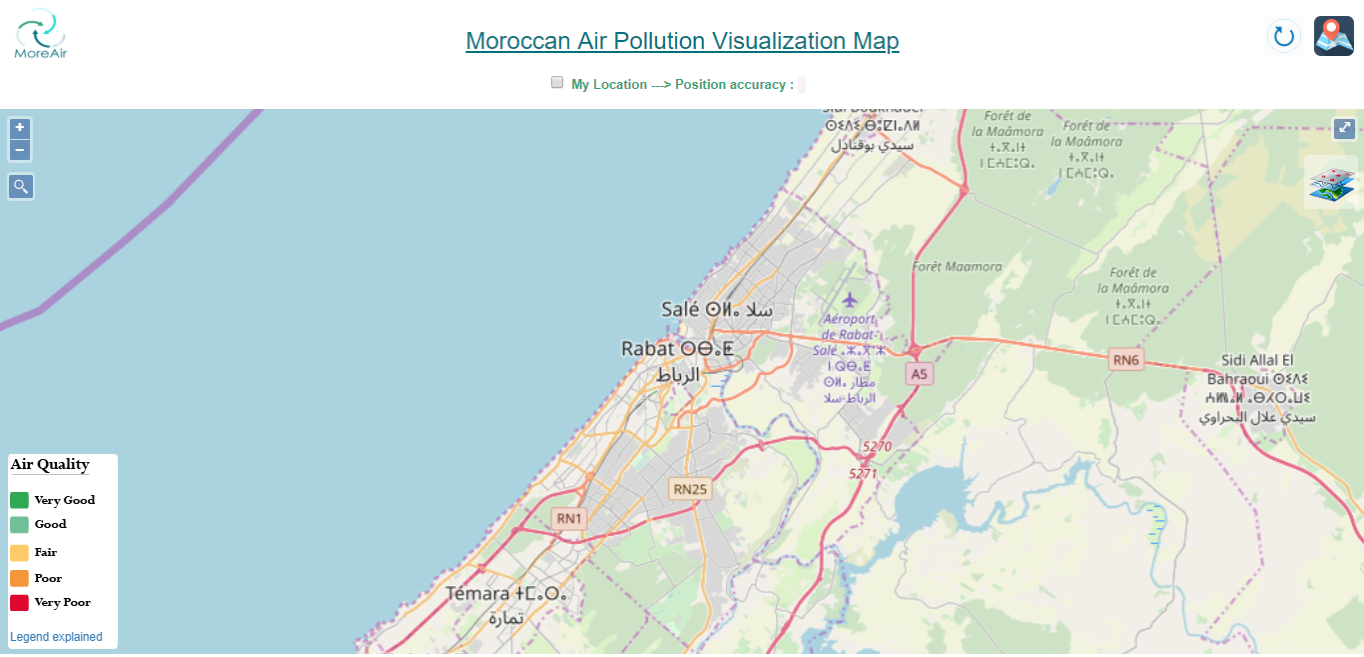
Moroccan Urban Air Quality Map.

In this section, we describe the developed Moroccan GIS for air pollution visualization. This GIS, called Moroccan Real-time Air Quality Visual Map, aims to provide real-time air quality information and forecasting for the region of Rabat-Salé-Témara first and then for all Moroccan cities in the future. It is developed using open source software. A screen capture of this map is shown in Figure 15.

**Figure 16:**
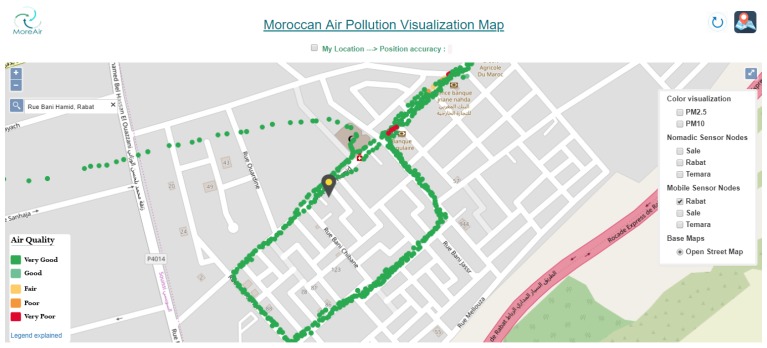
Mobile sensor nodes visualization in a neighborhood in Rabat.

The developed Air Pollution GIS offers several functionalities, namely it:

- allows a search by area address;
- shows the user’s position on the map and the associated accuracy
- allows a search by user GPS coordinates (latitude and longitude);
- shows the positions of the nomadic and mobile nodes on the map;
- shows a legend of air quality index with an explanation text;
- shows the pollutants’ visualization filter with color codes;
- Zooms on desired regions;

On the GIS, each measurement is accompanied by the date and time of the data collection. As shown in Figure 16, the mobile sensor measurements are represented with small discs on the map. Each disc on the map gives information about ${\mathrm{PM}}_{10}$ and ${\mathrm{PM}}_{2.5}$, humidity and temperature measurements at the location where the measurements were made. Furthermore, the disc’s color corresponds to the maximum of ${\mathrm{PM}}_{10}$ and ${\mathrm{PM}}_{2.5}$ concentrations. Moreover, a color visualization option is also available for each pollutant separately.

**Figure 17:**
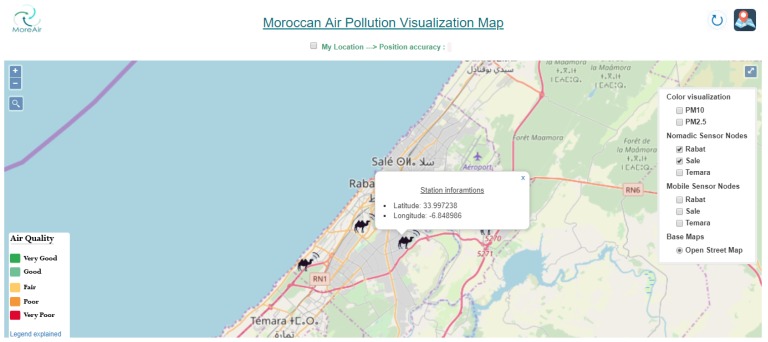
Visualization of nomadic sensor nodes and their location coordinates.

The nomadic air quality sensor nodes are presented on the map by camel icons at the locations where the sensor nodes were installed and kept for a period of time ranging from one week to a few months (see Figure 17). For each nomadic sensor node, information about the latitude and longitude of the node’s position are provided (see Figure 17).

**Figure 18:**
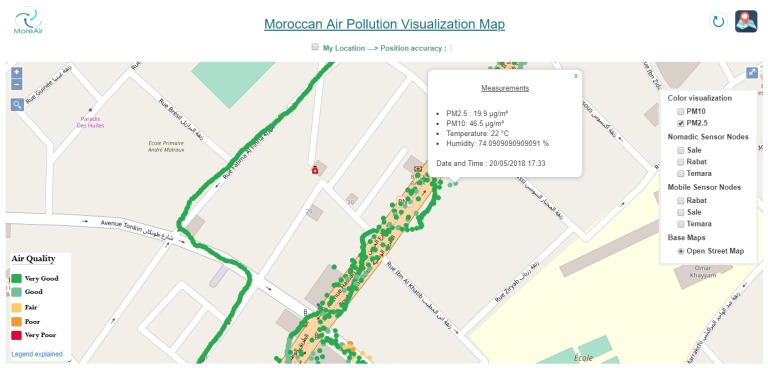
${\mathrm{PM}}_{2.5}$ measurements in a neighborhood of Rabat city.

Figure 18 shows an illustration example of ${\mathrm{PM}}_{2.5}$ measurements for a neighborhood in Rabat city. The colors of discs represent the air quality index (as described in Figure 9). Furthermore, real-time user positioning and its accuracy are also shown on the map. This allows the users to be informed about the air quality at their locations.

## 7. Conclusions and Future Work

In this paper, we have introduced MoreAir, a low-cost monitoring system that aims to provide agile, reliable and integrative air quality data collection. MoreAir is based on a customizable IoT platform and an innovative sensor deployment strategy that not only consists of nomadic and mobile sensor nodes, but also relies on a prior medical study to identify areas of high incidence of respiratory diseases. Using mobile sensor nodes, sources responsible for low air quality in different neighborhoods have been identified. Furthermore, this study has demonstrated the potential of using feature engineering in building reliable air pollution maps in urban areas. Indeed, in addition to traffic and meteorology related features, we have shown in this paper that in Moroccan neighborhoods other pollution sources may have a more significant impact on air quality than traffic; these are public baths, communal ovens, street food vendors and thrift shops. In this paper, we have also proposed a machine learning model to predict PM’s concentrations at a given location. While most of the existing models use meteorological features as predictors, we used both meteorological and traffic features to predict air quality. Random Forest is shown to provide better performance than Support Vector Machine and Multiple Linear Regression. Ongoing work consists of building larger datasets to developing spatio-temporal prediction models. Model-based spatial interpolation should yield a more reliable map of air pollution in urban areas. The combination of data generated by nomadic nodes (associated with low spatial coverage) and mobile nodes (associated with low time span coverage) is also being investigated to improve the accuracy of air pollution data.

## Figures and Tables

**Table 1:** Shortlist of sensor systems by pollutant, type, reference and cost.

Model	Pollutant	Type	Reference	Cost
MoreAir AQ-M	${\mathrm{PM}}_{10}$, ${\mathrm{PM}}_{2.5}$	OPC	-	$95
MoreAir AQ-N	${\mathrm{PM}}_{10}$, ${\mathrm{PM}}_{2.5}$	OPC	-	$95
Speck	${\mathrm{PM}}_{2.5}$	nephelometer	[25]	$150
Pure Morning P3	${\mathrm{PM}}_{2.5}$	OPC	[24]	$170
Dylos DC1100	${\mathrm{PM}}_{\text{2.5–0.5}}$	OPC	[26]	$300
AIRQino	${\mathrm{PM}}_{10}$, ${\mathrm{PM}}_{2.5}$	OPC	[27]	$1000
Met one -831	${\mathrm{PM}}_{10}$	OPC	[28]	$2000
SidePak AM510	${\mathrm{PM}}_{2.5}$	nephelometer	[29]	$3000
AQT-420	${\mathrm{NO}}_{2}$, $O_{3}$, ${\mathrm{PM}}_{10}$, ${\mathrm{PM}}_{2.5}$	electrochemical & OPC	[30]	$3700
AQMesh v.4.0	CO, ${\mathrm{NO}}_{2}$, NO, $O_{3}$, ${\mathrm{PM}}_{1}$, ${\mathrm{PM}}_{10}$, ${\mathrm{PM}}_{2.5}$	electrochemical & OPC	[31]	$10,000

**Table 2:** Data evaluation indicators.

	$R^{2}$	MAE
${\mathrm{PM}}_{10}$	0.84	3.97
${\mathrm{PM}}_{2.5}$	0.81	1.03

**Table 3:** Pollution sources and pollution concentrations measured in their vicinity.

Pollution Sources	PM_10_ (μg/m^3^)	PM_2.5_ (μg/m^3^)
Public Baths and Ovens	400	35
Open-Air Thrift Shops	200	150
Street Food Vendors	100	70
Traffic	80	53

**Table 4:** Results of the prediction algorithms.

	PM_10_		PM_2.5_	
	RMSE	R^2^	RMSE	R^2^
MLR	38.86	0.22	13.92	0.42
SVR	14.61	0.39	9.25	0.47
Random Forest	13.63	0.57	7.76	0.63
